# *Lactobacillus* Suppresses Tumorigenesis of Oropharyngeal Cancer *via* Enhancing Anti-Tumor Immune Response

**DOI:** 10.3389/fcell.2022.842153

**Published:** 2022-03-01

**Authors:** Ke-Ke Wang, Kai-Yue He, Jing-Yu Yang, Meng-Jie Liu, Jin-Rong Guo, Ji-Yong Liang, Jin-Hua Wang, Zhi-Xiang Xu, Yong-Ping Jian

**Affiliations:** ^1^ School of Life Sciences, Henan University, Kaifeng, China; ^2^ Jiangsu Cancer Hospital, Jiangsu Institute of Cancer Research, and The Affiliated Cancer Hospital of Nanjing Medical University, Nanjing, China; ^3^ Department of Neuro-Oncology, The University of Texas MD Anderson Cancer Center, Houston, TX, United States

**Keywords:** oropharyngeal cancers, microbiota dysbiosis, lactobacillus, acetate, antitumor immune response

## Abstract

Deficiency in T cell-mediated adaptive immunity, such as low CD8^+^ T cell infiltration, inhibits the immune surveillance, promotes malignant transformation, and facilitates tumor growth. Microbiota dysbiosis diminishes the immune system and contributes to the occurrence of cancer. However, the impact of oral dysbiosis on the occurrence and molecular mechanisms of oropharyngeal cancer (OPC) remains largely unknown. In the current study, we used 4-nitroquinoline-1-oxide (4NQO) to mimic tobacco-related carcinogenesis to generate a murine OPC model and determine the role of microbiota changes in OPC tumorigenesis. Our results showed that the oral flora composition of mice was deregulated during the tumorigenesis of OPC. The abundance of *Streptococcus*, *Veillonella*, *Muribacter*, *Rodentibacter*, and *Gemella* was increased, whereas the dominant genus *Lactobacillus* was gradually decreased with disease progression. We further demonstrated that infiltration of CD8^+^ T lymphocytes was markedly reduced due to the reduction of *Lactobacillus.* Supplementation of *Lactobacillus* increased the infiltration of CD8^+^ T cells, promoted the expression of IFN-*γ* and granzyme B, and lessened the OPC progression. Analyzing the metabolites of the *Lactobacillus*, we demonstrated that *Lactobacillus* enhanced the anti-tumor immune response by producing acetate in OPC development. Administration of acetate to mice could increase the expression of IFN-*γ* and IFN-*γ*-inducible chemokines in tumor tissues by activating GPR43 to promote the infiltration of CD8^+^ T lymphocytes and substantially delay the development of OPC. Together, our data suggest that dysbiosis of oral microbiota promotes the tumorigenesis of OPC through downregulation of cytotoxic T lymphocytes. *Lactobacillus* and its metabolite acetate improve the tumor microenvironment, which could be applied in the treatment of OPC.

## Introduction

Oropharyngeal cancer (OPC) is a universal malignant tumor in the head and neck with a high incidence in patients with a history of smoking. Ninety percent of OPCs are squamous cell carcinoma (Parkin et al., 1999). Although surgery, radiotherapy and chemotherapy have made significant progresses in the treatment of OPC, the 5-year survival rate of patients is only about 30% (Rusthoven et al., 2008; Siegel et al., 2014). Currently, tumor immunotherapy shows continuously clinical response and is triggering a shift in cancer treatment. CD8^+^ T cells play a dominant role in tumor immunity. Less infiltration or dysfunction of CD8^+^ T cells in the tumor microenvironment (TME) has led to poor clinical outcomes for many cancers (Yu and Fu, 2006; Houot et al., 2015; He et al., 2021). Therefore, promoting the infiltration and function of CD8^+^ T cells in TME is beneficial to improve the efficacy of cancer treatment.

The microbiota plays a vital role in human health. Studies have shown that 15–20% of cancers are caused by microbial dysbiosis (D'Souza et al., 2007). Katz et al. found a large number of periodontal pathogens-*Porphyromonas gingivalis* (*P. gingivalis*) in gingival squamous cell carcinoma (GSCC) tissues (Katz et al., 2011; Ritter and Greten, 2019), in which microorganism-mediated chronic inflammation plays a role in promoting disease progression (Bhatt et al., 2017). Recent studies on mice and humans have shown that gut microbiome modulates the anti-tumor efficacy in chemotherapy and immunotherapy by shaping host immunity (Iida et al., 2013; Gopalakrishnan et al., 2018; Matson et al., 2018; Routy et al., 2018; Tanoue et al., 2019; Jian et al., 2021). Compared with the untreated group, cancer patients treated with antibiotics had a lower response to anti-PD-1 immunotherapy. Restructure of sterile mice with feces from anti-PD-1 responding patients improves tumor control and T cell responses (Routy et al., 2018). The intestinal microbiota regulates dendritic cells and CD4^+^ T cells to enhance cancer immune surveillance and advance therapeutic effects (Iida et al., 2013; Vétizou et al., 2015; Gopalakrishnan et al., 2018; Matson et al., 2018; Routy et al., 2018; Tanoue et al., 2019; Jian et al., 2021). However, it is still not fully understood how the oral microbiota changes and whether the oral microbiota could regulate the tumor-infiltrating CD8^+^ T cell responses in OPC.

The microbiota synthesizes multiple metabolites, which may affect the development of cancer and impact systemic immune responses (Levy et al., 2017; Mariño et al., 2017; Zitvogel et al., 2017). Short-chain fatty acids (SCFAs), including acetic acid, butyrate, and propionic acid, are produced by bacterial fermentation of dietary fiber (Koh et al., 2016). The SCFA-mediated activation of G protein-coupled receptor 43 (GPR43) promotes the expression of IFN. GPR43 deficiency leads to decreased IFN production (Antunes et al., 2019). IFN-*γ*-inducible CXCL9, CXCL10, and CXCL11 are chemokines attracting cytotoxic T cells to infiltrate into tumor tissues and exert anti-tumor effects (Gandhi et al., 2021). In addition, SCFAs also affect autoimmune CD8 +T cell response to prevent diabetes (Tan et al., 2017). These data prompted us to investigate whether the oral microbial metabolites could promote anti-cancer immunity and improve the therapeutic effect.

In order to investigate the effects of oral flora in OPC development, we collected pharyngeal tissues from mice. Through 16S rRNA sequencing, we analyzed the alterations in the composition and diversity of the flora and studied the impact of microbiota changes on the immune system. We identified that *Lactobacillus* is the dominant genus in the oral cavity and *Lactobacillus* metabolites slowed the OPC progression by activating GPR43 to increase the number of tumor-infiltrating CD8^+^ T cells, indicating that supplementation of specific microbiota and metabolite may have an impact on future cancer therapy.

## Materials and Methods

### Establishment of Animal Model

Healthy BALB/c male mice were housed in animal barrier facility in Henan University on a 12 h light-dark cycle with food and water available ad libitum. Mice were randomly assigned into two cages in each group for collecting feces and recording survival. We randomly divided BABL/c male mice into an experimental group (*n* = 20) provided with drinking water containing 100 mg/L 4NQO (sigma, United States) and into a control group (*n* = 20) provided with drinking water without 4NQO (Bouaoud et al., 2021). Five mice were randomly sacrificed every 4 weeks, continuously for 16 weeks. Experiments involving mice were under the regulation of ethical requirements and approved by the institutional animal care and use committee (IACUC) of Henan University in China.

### Glossopharyngeal Tissue Collection

The mice were sacrificed by cervical dislocation and then dissected immediately. The mouse glossopharyngeal tissue was taken out and divided longitudinally from the midline, and washed with normal saline. One part was subjected to 16S rRNA sequencing. The other one was fixed in 10% neutral formalin buffer and dehydrated by ethanol gradient after 24 h, followed by embedded in conventional paraffin, sectioned at 2 μm, stained with HE, and observed under an optical microscope.

### Application of *Lactobacillus* in Mice

*Lactobacillus* was purchased from American Type Culture Collection (202195) and cultured in *Lactobacillus* MRS broth (Panigrahi et al., 2017). *Lactobacillus* was inactivated by pasteurization for 30 min at 70°C (Wang et al., 2020). After pasteurization, no viable *Lactobacillus* could be recovered in culture. 2 × 10^9^ CFU of *Lactobacillus* in 0.2 ml PBS or PBS alone was dripped into the mouse mouth for 16 weeks (Si et al., 2021).

### HE Staining

A total of 2-μm sections of glossopharyngeal tissue from different groups were processed for HE staining for histopathology. Histopathological examination was performed by three experienced oral pathologist in a blind manner basically according to the criteria described by Kramer et al. (Kramer et al., 1978).

### Diagnosis and Grading Criteria for Epithelial Dysplasia

Epithelial dysplasia was diagnosed as previous described (Stanley et al., 1992). 1) The polarity of epithelial basal cells disappeared; 2) More than one layer of basal-like cells appeared; 3) The nucleo-plasma ratio of cells increased; 4) The epithelial spikes were droplet; 5) Epithelial hierarchy was disordered; 6) Mitotic index increased with a few abnormal mitosis; 7) Mitosis appeared in the superficial half of the epithelium; 8) Cellular atypia; 9) Nuclear hyperchromia; 10) Nucleolar enlargement; 11) Decreased intercellular cohesion; 12) Keratinization of single or clustered cells in the spinous layer. Tissues with two items of the above conditions are mild dysplasia. Those with three to four items are moderate dysplasia, and focuses with more than five, or epithelial hierarchy severely disordered, are severe dysplasia. Carcinoma *in situ* refers to severe dysplasia involving the whole layer of epithelium, but not yet invading the basement membrane and growing downward. Those who break through the basement membrane are invasive cancer (Stanley et al., 1992).

### Short-Chain Fatty Acids Detection by Gas Chromatography–Mass Spectrometry Quantitation

SCFAs in the glossopharyngeal tissue and serum of mice were quantified as described previously (Guo et al., 2020) with minor modifications. Appropriate amount of sample was added in a 2-ml centrifuge tube followed by mixing with 50 μL of 15% phosphoric acid, 10 μL of 75 μg/ml internal standard (isohexanoic acid) solution, and 140 μL ether for 1 min. The mixture was then centrifuged at 12,000 rpm for 10 min at 4°C, and the supernatant was used for analysis with the Chromatographic Agilent HP-INNOWAX capillary column (30 m*0.25 mm ID*0.25 μm). Split injection volume was 1 μL and split ratio was 10:1. The carrier gas was helium and the carrier gas flow rate was 1.0 ml/min.

### Immunohistochemistry

After deparaffinizing, the 2-μm glossopharyngeal tissue section was hydrated in gradient ethanol. For antigen retrieval, slides were immersed in 0.01 M sodium citrate buffer and heated for 30 min. After inactivating endogenous peroxidase with 3% hydrogen peroxide and blocking with goat serum albumin, the sections were incubated with anti-CD8 antibody (1:200, Abcam, United Kingdom) at 4°C for overnight. The slides were incubated with biotinylated secondary antibodies. Then the sections are incubated with peroxidase-streptavidin and stained with 3,3′-diaminobenzidine tetrahydrochloride (DAB) for 30 s. Finally, the nuclei were counterstained with hematoxylin. As a negative control, tissue sections were processed in parallel by incubating with PBS instead of primary antibody.

The semi-quantitative assessment of IHC staining was reviewed by three different pathologists and classified as negative, weak, medium, or strong. To determine the H score, antibody-stained tissue was scored by calculating the product of the percentage of cells staining for each intensity level and the intensity level (0, negative; 1+, weak; 2+, medium; 3+, strong). Then the individual intensity level scores are added together to calculate the H score (Sun et al., 2020).

### 16S rRNA Sequencing

DNA in the glossopharyngeal tissue of mice was extracted with a DNA extraction kit (GenElute™ Stool DNA Isolation Kit, Sigma-Aldrich), and the purity and concentration of sample DNA were detected by ultraviolet spectrophotometer. The sample DNA was subjected to 1% agarose gel electrophoresis to determine the integrity of DNA. After DNA was qualified, PCR amplification was carried out, and 16S rRNA gene V3-V4 region was amplified. PCR amplification primers for V3-V4 region were 338 F (ACT​CCT​ACG​GGA​GGC​AGC​AG) and 806 R (GGACTACHVGGGTWTCTAAT) (Liu et al., 2016). The amplified sample was validated by 2% agarose gel electrophoresis, and the target band size of PCR products was confirmed before any subsequent experiments were performed. The high-throughput sequencing and data analysis involved were completed by Shanghai Meiji Biology Company (Shanghai, China).

### Cytokine Analysis

The secretion of Granzyme B, IFN-*γ* and IFN-*γ*-inducible chemokines from tumors was detected by ELISA kits (R&D Systems, Minneapolis, MN). After the mouse was euthanized, the glossopharyngeal tumors were isolated from the mouse immediately. The glossopharyngeal tumors were mechanically homogenized in PBS containing protease inhibitors (10 ug/mL aprotinin, 10 ug/mL leupeptin and 10 ug/mL pepstatin). After homogenization, Triton X-100 (Applichem, Darmstadt, Germany) was added to a final concentration of 1%. The samples were frozen at −80°C, thawed and centrifuged at 10,000 × g for 5 min to remove cell debris. The supernatant of tumors homogenate was examined for cytokine production by ELISA according to the manufacturer’s instructions. The homogenates from three tumors per group were pooled for the analysis. Signal intensity was calculated using Image J (Aindelis et al., 2020).

### Statistical Analysis

Statistical analysis was performed in SPSS 21.0 program. The quantitative data were expressed as mean ± standard deviations (SD) and were analyzed by one way ANOVA. *p* < 0.05 was considered significant. (∗*p* < 0.05; ∗∗*p* < 0.01; ∗∗∗*p* < 0.001; ns, no significant difference).

## Results

### Development of Premalignant and Oropharyngeal Cancer in 4NQO-Treated Mice in a Time-dependent Manner

In order to establish an OPC mice model, we treated BABL/c male mice with 4NQO in drinking water for 16 weeks. Five mice were randomly euthanized every 4 weeks (Supplementary Figure S1A). At the beginning of the experiment, there was no significant difference in body weight between control and 4NQO-treated mice (*p* > 0.05). At the end of 16 weeks, body weights of 4NQO-treated mice decreased as compared with those in control mice (*p* < 0.05) (Supplementary Figure S1B). With the prolongation of 4NQO treatment, oropharyngeal tissues of mice displayed a typical progression from mild, moderate, and severe dysplasia to OPC (Supplementary Figure S1C).

### Changes of Oral Flora Composition and Diversity in Mice With Dysplasia and Oropharyngeal Cancer

**FIGURE 1:**
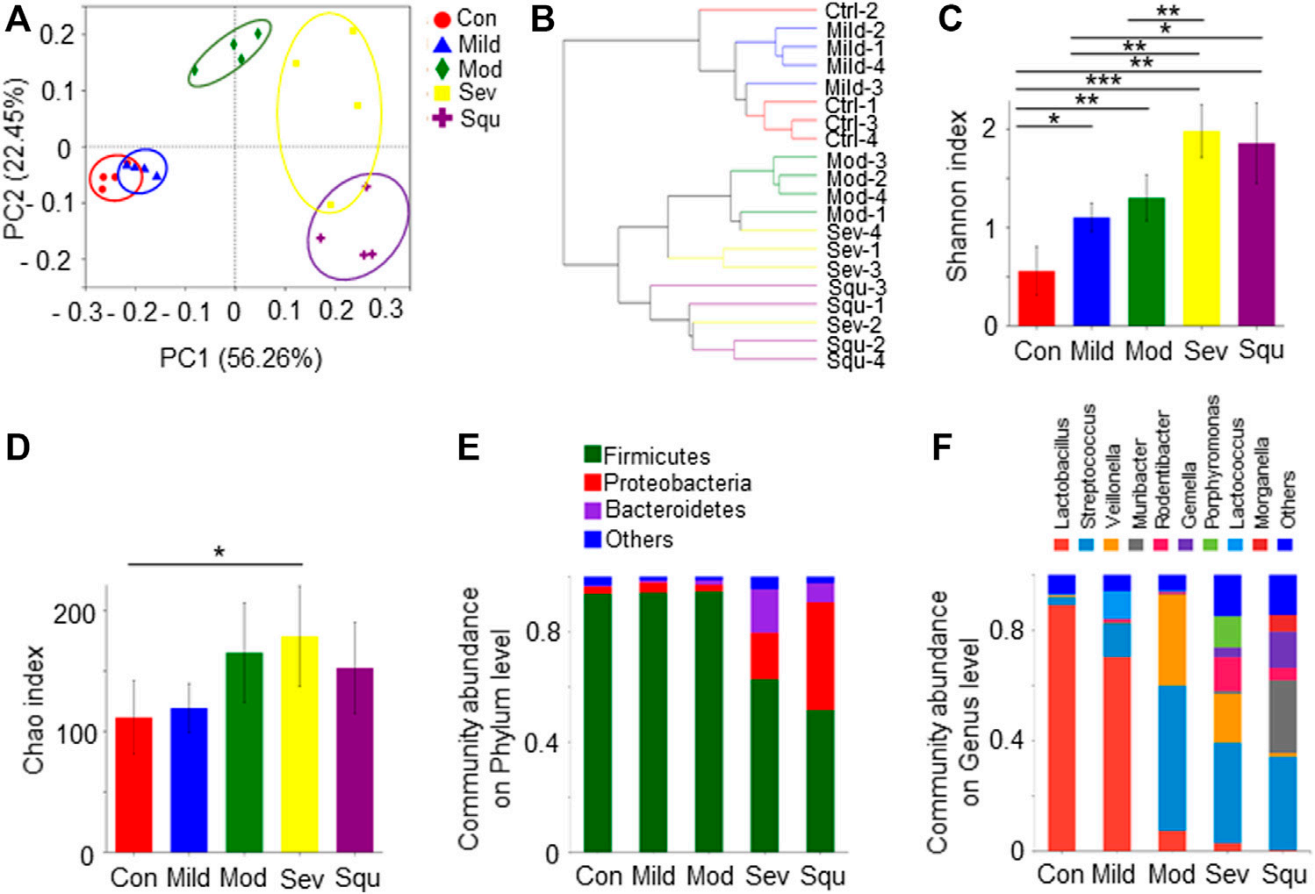
Composition and diversity of oral flora in mice with dysplasias and OPC. SPF BALB/C mice were treated with or without 100 mg/L 4NQO in drinking water for 0–16 weeks. After the treatment, mice were sacrificed and the glossopharyngeal tissues were collected. Total DNAs were isolated from the glossopharyngeal tissues of mice as described in Materials and Methods and subjected to 16S rRNA sequencing. **(A)** PCA of oral flora. **(B)** Hierarchical clustering maps of oral flora. **(C)** Shannon index of oral flora. **(D)** Chao index of oral flora. **(E)** Composition of oral flora on phylum level. **(F)** Composition of oral flora on genus level. Data represent mean ± SEM. *p < 0.05, **p < 0.01, ***p < 0.001, *n* = 4, compared with control.

To determine the alteration of oral microbiome in the development of OPC, we examined dynamics of the oral microbiota throughout the formation of OPC in our animal model. We performed a 16S rRNA sequencing on the oral flora of mice. PCA analysis of the sequencing results showed that control mice and dysplasia or OPC mice bore different clusters. With the extension of dysplasia, a farther distance in the composition of the oral flora from control mice were observed, indicating that components of microbiota were altered during OPC development (Figure 1A). The hierarchical clustering analysis also yielded parallel results (Figure 1B), supporting our notion that oral microbiota composition of mice with dysplasia and OPC was different from that of control animals.

We further examined oral microbiota diversity of the mice with dysplasia and OPC. Shannon and Chao indexes were used to measure the diversity and abundance of microbiota. We found that alpha diversity and abundance of the oral flora in OPC mice were significantly different from those in control mice (*p* < 0.05). As progression of atypical hyperplasia to OPC, the diversity and abundance of oral microflora in mice were significantly higher than those in control mice (Figures 1C,D). At the phylum level, the oral bacteria of control mice are mainly composed of *Firmicutes*, *Proteobacteria*, and *Bacteroidetes*. *Firmicutes* gradually decreased with the aggravation of atypical hyperplasia, accompanied by the increase of *Proteobacteria* (Figure 1E). At the genus level, the abundance of *Streptococcus*, *Veillonella*, *Muribacter*, *Rodentibacter*, and *Gemella* was increased in dysplasia or OPC mice, whereas the abundance of *Lactobacillus* was decreased (Figure 1F). *Lactobacillus* was the dominant genus, which gradually decreased with worsening of atypical hyperplasia (Figure 1F). These results strongly suggest that OPC tumorigenesis is related to the dysbiosis of the oral microbiota, as highlighted by significant shifts in bacterial populations from a broad range of taxonomic groups.

### Abundance of *Lactobacillus* Was Reduced in Mice With Oropharyngeal Cancer

**FIGURE 2:**
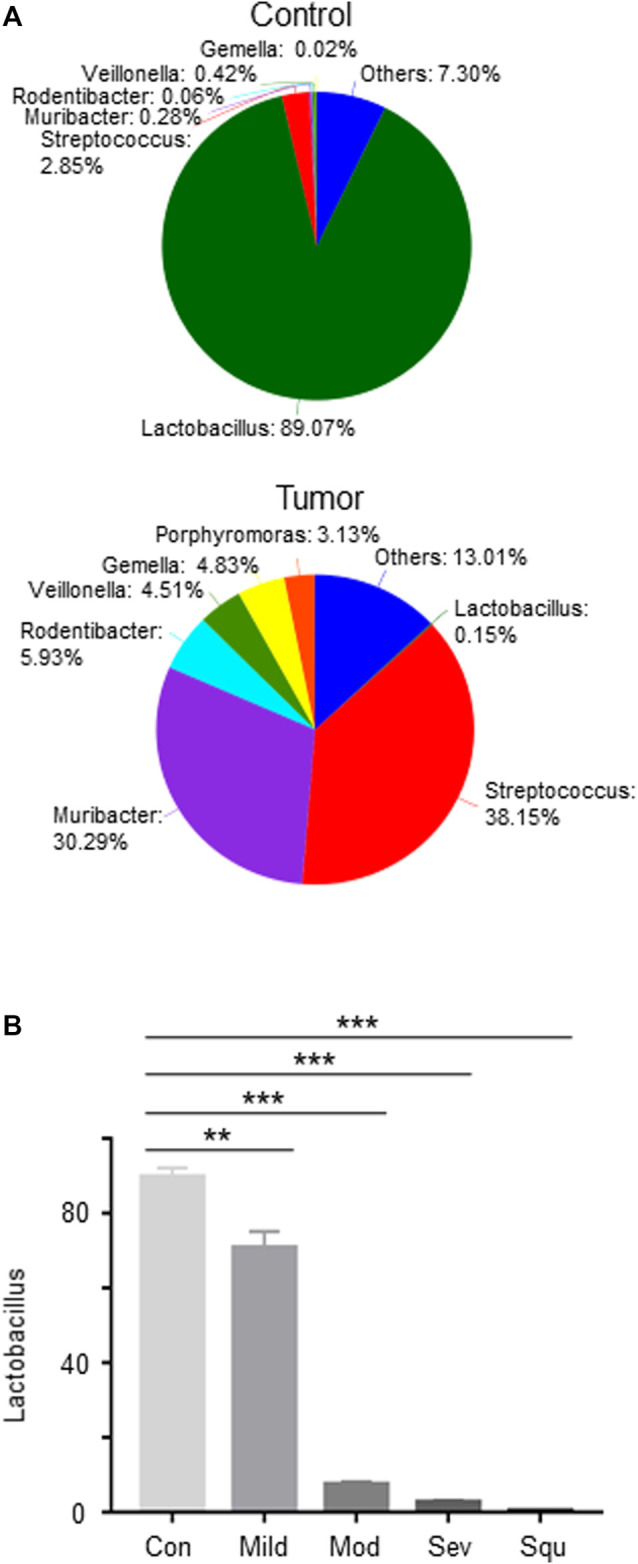
Abundance of *Lactobacillus* is reduced in mice with dysplasias and OPCs. Mice were treated as described in Figure 1. Glossopharyngeal tissues of mice with dysplasias and OPC were collected. Total DNAs were isolated from the glossopharyngeal tissues of mice as described in Materials and Methods and subjected to 16S rRNA sequencing. **(A)** Pie analysis of oral flora in control and OPC mice. **(B)** The proportion of *Lactobacillus* in the glossopharyngeal tissues of mice with dysplasias and OPCs. Data represent mean ± SEM. **p < 0.01, ***p < 0.001, *n* = 3, compared with control.

To further explore the impact of oral microbiota on tumorigenesis of OPC, we analyzed the differences in oral flora between control mice and tumor-producing mice. We analyzed the proportions of different microorganisms through Pie diagrams. As compared with control mice, OPC mice showed an upregulated abundance of *Muribacter*, *Rodentibacter*, *Veillonella*, *Gemella*, *Streptococcus* and *Porphyromoras*, and a downregulated abundance of *Lactobacillus* (Figure 2A). More importantly, as compared with control mice, *Lactobacillus* gradually decreased with worsening of atypical hyperplasia, being the highest reduction in OPC mice (Figure 2B), indicating that *Lactobacillus* may be involved in the pathogenesis of OPC.

### Supplement of *Lactobacillus* Slows Down Oropharyngeal Cancer Development

**FIGURE 3:**
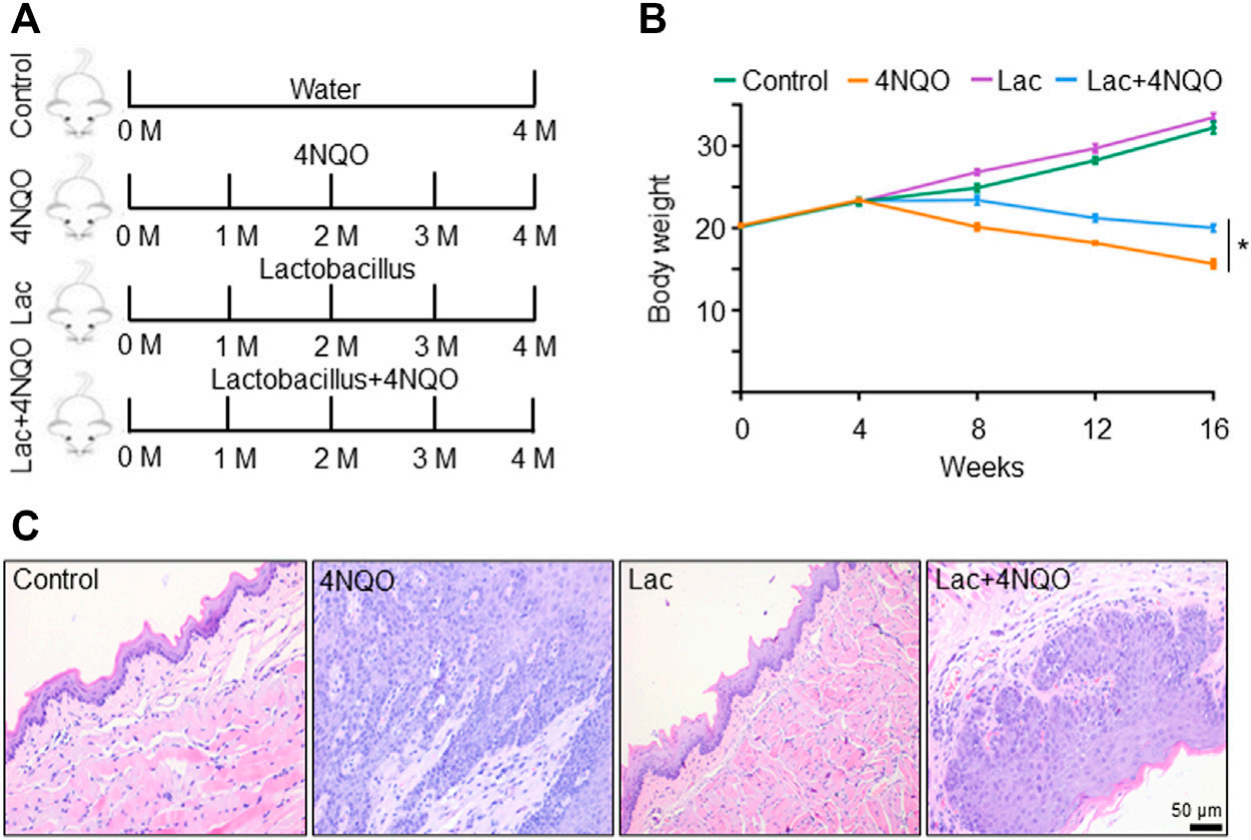
Supplement of *Lactobacillus* slows down OPC tumorigenesis. Mice were treated as described in Figure 1. 2 × 10^9^ CFU of *Lactobacillus* in 0.2 ml PBS or PBS alone was dripped into the mouth of mice treated with 4NQO for 16 weeks. Five mice were randomly sacrificed every 4 weeks continuously for 16 weeks. **(A)** Experimental procedures. **(B)** Body weight of mice. **(C)** HE staining of glossopharyngeal tissue. Data represent mean ± SEM. *p < 0.05, *n* = 5, compared with 4NQO alone.

Since *Lactobacillus* is significantly reduced in OPC tissues, we inquired whether supplement of *Lactobacillus* could slow down OPC progression. We treated mice with *Lactobacillus* and 4NQO simultaneously for 16 weeks (Figure 3A), and found that application of *Lactobacillus* significantly alleviated 4NQO-induced body weight reduction of the mice (Figure 3B). Histologically, oropharyngeal and tongue tissues in control- and *Lactobacillus-*treated mice were normal. Those in mice treated with 4NQO alone experienced typical pathological changes from normal to mild, moderate, and severe dysplasia to early invasive carcinoma. Application of *Lactobacillus* to 4NQO-treated mice markedly delayed the progression of oropharyngeal tissue dysplasia with only moderate to severe dysplasia being observed at the end of 16w-exposure (Figure 3C), indicating that *Lactobacillus* supplementation slows down the carcinogenesis of 4NQO.

### Short-Chain Fatty Acids, in Particular Acetate, Contribute to *Lactobacillus*-Induced Anti-Tumor Effects

**FIGURE 4:**
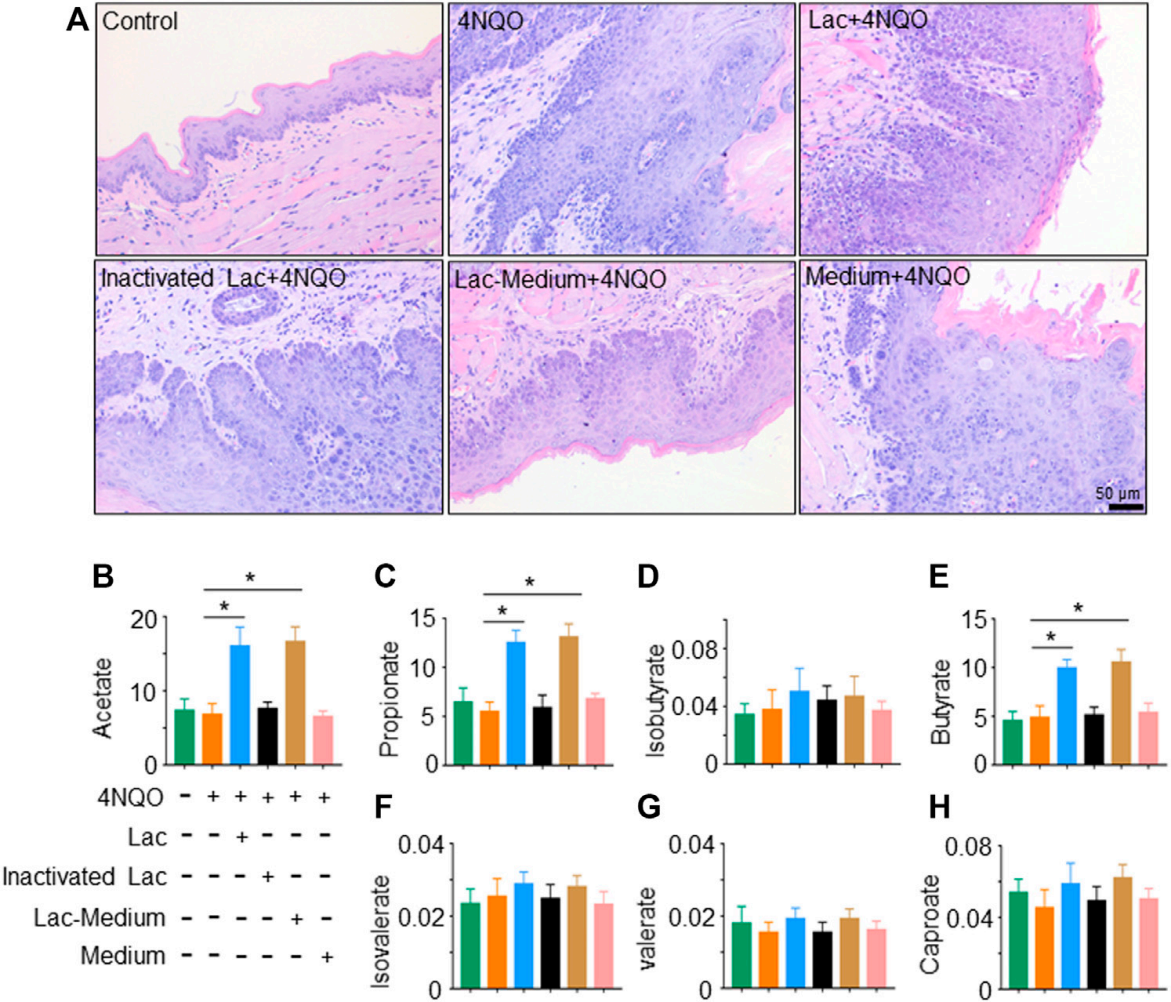
Viable *Lactobacillus* is necessary for delaying OPC tumorigenesis. *Lactobacillus* was inactivated by pasteurization for 30 min at 70°C. After pasteurization, no viable *Lactobacillus* could be recovered in culture. 2 × 10^9^ CFU of *Lactobacillus*, inactivated *Lactobacillus*, *Lactobacillus* culture supernatant (Lac-Medium) or Medium alone was dripped into mouth of mice treated with 4NQO for 16 weeks. SCFAs in glossopharyngeal tissue of the mice were quantified as described in Materials and Methods. **(A)** HE staining of glossopharyngeal tissue. **(B–H)** Levels (µg/ml) of acetate, propionate, isobutyrate, butyrate, isovalerate, valerate, and caproate in glossopharyngeal tissue of the mice. Data represent mean ± SEM. *p < 0.05, *n* = 5, compared with 4NQO alone.

**FIGURE 5:**
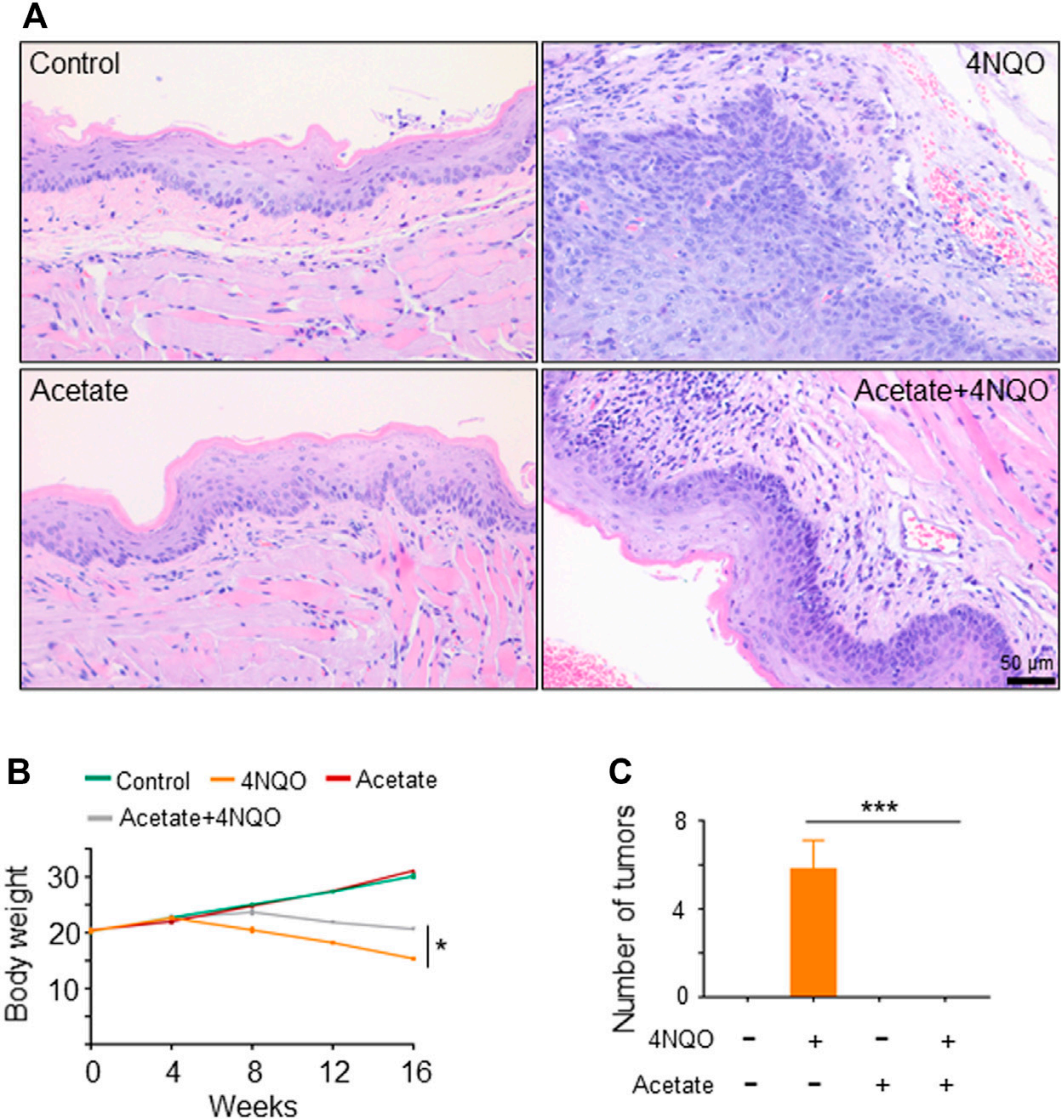
Metabolites of *Lactobacillus,* in particular short-chain fatty acids (SCFAs), contribute to *Lactobacillus*-induced anti-tumor effects. SPF BALB/C mice were treated with 150 mM acetate and/or 4NQO for 4 months. After the treatment, the mice were sacrificed and the glossopharyngeal tissue of mice was collected for histological staining. **(A)** HE staining of glossopharyngeal tissue. **(B)** Body weight of mice. **(C)** Number of tumors in each mouse. Data represent mean ± SEM. *p < 0.05, ***p < 0.001, *n* = 5, compared with 4NQO.

To determine how *Lactobacillus* slows down the development of OPC, we tested whether *Lactobacillus* acts through its own components or metabolites to exert anti-tumor effects. We found that viable, but not pasteurization-inactivated, *Lactobacillus* or its culture supernatant markedly delayed the onset of OPC induced by 4NQO (Figure 4A). It is reported that *Lactobacillus* produces short-chain fatty acids (SCFAs), which reduce the expression and function of intestinal P-glycoprotein (P-gp) and upregulate breast cancer resistance protein (BCRP), leading to tumor suppression *via* the inhibition of HDAC/NF-κB and activation of PPAR*γ* (Xie et al., 2021). To determine whether SCFAs mediate the anti-tumor effect of *Lactobacillus*, we analyzed SCFA contents in the glossopharyngeal tissue of mice treated with or without *Lactobacillus* by gas chromatography–mass spectrometry. We found that levels of acetate, propionate and butyrate in the glossopharyngeal tissue of *Lactobacillus*-treated and *Lactobacillus* supernatant-treated mice were substantially increased, whereas no difference was identified in the levels of isobutyrate, isovalerate, valerate, and caproate (Figures 4B–H). Compared with mice treated with the medium, acetate, propionate and butyrate in the serum of the mice treated with *Lactobacillus* supernatant increased significantly, and there was no statistical difference in the remaining SCFAs (Supplementary Figures S2A–G). Among SCFAs tested, acetate possessed a highest concentration (Figures 4B–H; Supplementary Figures S2A–G). To assess the role of SCFA(s) secreted by *Lactobacillus* in slowing the progression of OPC, we evaluated the anti-tumor effect of acetate. We found that treatment with 150 mM acetate (Xie et al., 2021) in drinking water for 16 weeks significantly slowed down 4NQO-induced OPC development (Figure 5A), increased the weight of mice (Figure 5B) and reduced the number of tumors (Figure 5C). Together, our results support the notion that acetate is a critical mediator in *Lactobacillus*-reduced the OPC progression.

### Metabolites of *Lactobacillus* Enhance Anti-Tumor Immunity Against Oropharyngeal Cancer in Mice

**FIGURE 6:**
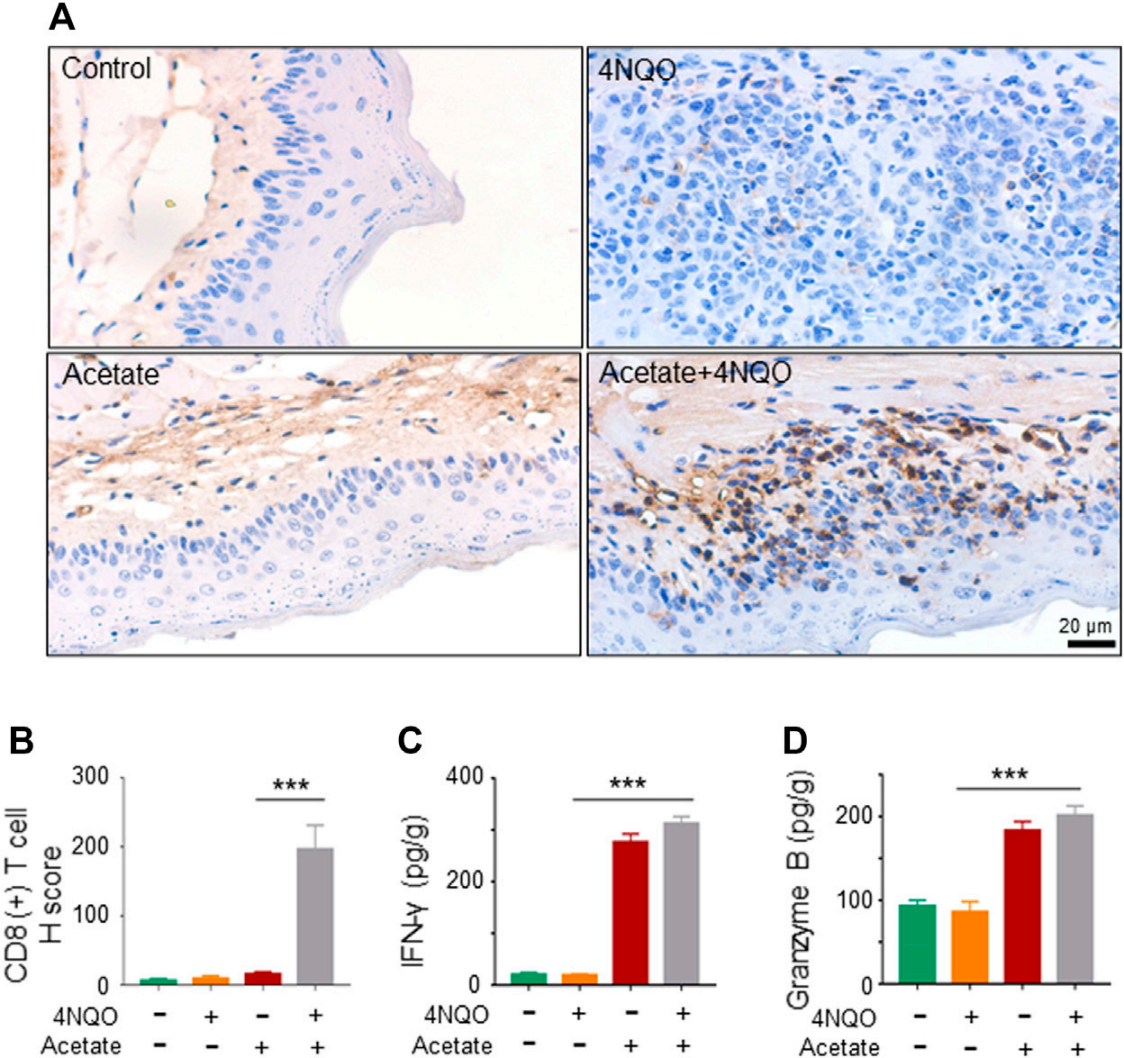
Supplement of acetate enhances murine anti-tumor immunity against OPC. The mice were treated as described in Figure 5. Glossopharyngeal tissue was collected for histological staining and cytokine detection. The secretion of Granzyme B and IFN-*γ* from tissues was detected by ELISA. **(A,B)** IHC staining of CD8^+^ T cells **(A)** and its quantitation **(B)** were performed in glossopharyngeal tissues. **(C,D)** The level of IFN-*γ* **(C)** and Granzyme B **(D)** in the glossopharyngeal tissue. Data represent mean ± SEM. ***p < 0.001, *n* = 5, compared with 4NQO.

The infiltration and activation of immune cells in the tumor microenvironment affect the occurrence and development of tumors, especially that lower CD8^+^ T cell infiltration directly promotes tumor growth (He et al., 2021). T cell-mediated adaptive immunity protects against the malignant transformation. Oral microecological dysbiosis diminishes the immune system and hence contributes to the occurrence of oral cancer and OPC (Bhatt et al., 2017). To examine the effect of acetate on immune microenvironment of OPC, we analyzed CD8^+^ T lymphocytes in 4NQO-induced OPC treated with or without acetate*.* We observed an increased infiltration of CD8^+^ cytotoxic T cells into the sublesional areas of mice that received a simultaneous treatment of acetate and 4NQO, as compared with lesions in mice treated with 4NQO alone (Figures 6A,B). In addition, levels of IFN-*γ* and granzyme B were significantly higher in OPCs from mice fed with acetate and 4NQO than those in mice treated with 4NQO alone (Figures 6C,D). With the increase of CD8^+^ cytotoxic T cells in the lesions from mice treated with acetate, lower degree of dysplasia in oropharyngeal tissues of 4NQO-treated mice was observed, supporting our notion that *Lactobacillus* secretes acetate to slow down the OPC progression by enhancing the anti-tumor immunity.

### *Lactobacillus* Metabolites Activate GPR43 to Enhance Anti-Tumor Immunity *via* Increasing the Production of IFN-*γ*-Inducible Chemokines

**FIGURE 7:**
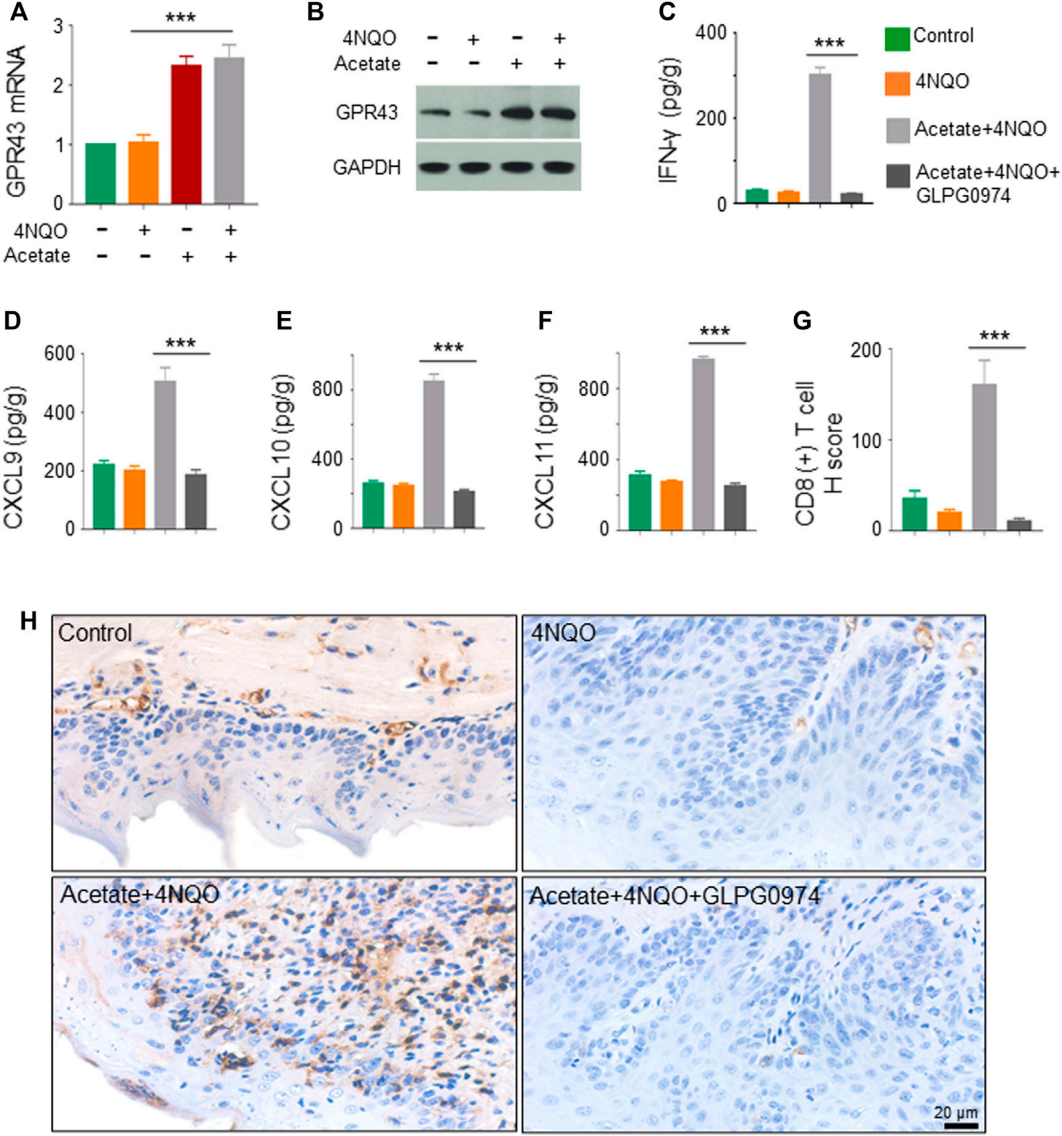
*Lactobacillus* metabolites promote the expression of IFN-inducible chemokines through activating GPR43. The mice were treated as described in Figure 5. GPR43 inhibitor GLPG0974 (HY-12940, MCE, Monmouth, NJ, United States) was dripped into the oral cavity of mice at a dose of 10 mg/kg, once a week for 16 consecutive weeks. Glossopharyngeal tissue was collected for histological staining and cytokine detection. The secretion of IFN-*γ*, CXCL9, CXCL10 and CXCL11 from tissues was detected by ELISA. **(A,B)** Transcription **(A)** and expression **(B)** of GPR43 in the glossopharyngeal tissue. **(C–F)** The level of IFN-*γ*, CXCL9, CXCL10 and CXCL11 in the glossopharyngeal tissue. **(G,H)** CD8^+^ T cells in the glossopharyngeal tissue by IHC staining. IHC quantitation **(G)** and representative staining of CD8^+^ T cells in glossopharyngeal tissue **(H)** were presented. Data represent mean ± SEM.***p < 0.001, *n* = 5, compared with Acetate+4NQO + GLPG0974.

**FIGURE 8:**
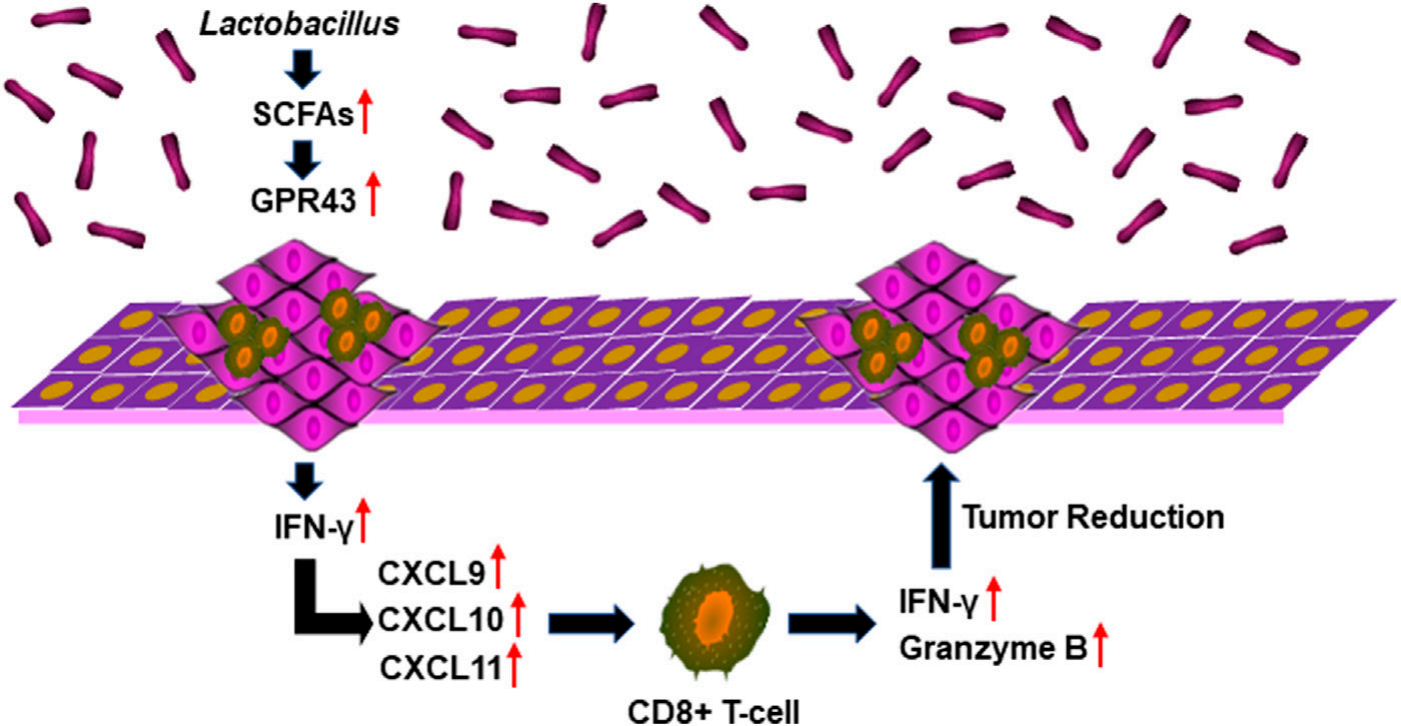
*Lactobacillus* metabolites suppress the development of OPC through anti-tumor immunity. *Lactobacillus* metabolites increased the expression of IFN-*γ* and IFN-*γ*-inducible chemokines CXCL9, CXCL10 and CXCL11 in oropharyngeal tissues by activating GPR43, thereby promoting the infiltration of CD8^+^ T cells and the secretion of IFN-*γ* and Granzyme B and inhibiting the OPC development.

SCFA-mediated activation of G protein-coupled receptor 43 (GPR43) promotes the expression of IFN. GPR43 deficiency leads to decreased IFN production stimulated by SCFAs (Antunes et al., 2019). We found that the transcription and expression of GPR43 in OPC tumors in mice treated with acetate and 4NQO were strikingly increased as compared with those in mice treated with 4NQO alone (Figures 7A,B). To determine the role of GPR43 in the production of IFN-*γ* in OPC tissues from acetate-treated mice, we treated mice with GPR43 inhibitor GLPG0974 once a week for 16 weeks, during which acetate and 4NQO were administrated. As expected, treatment of acetate significantly elevated the level of IFN-*γ* in OPC tissues of mice. However, IFN-*γ* expression was significantly reduced in OPC tissues from Acetate+4NQO mice with the addition of GLPG0974 (Figure 7C). IFN-*γ*-inducible CXCL9, CXCL10 and CXCL11 are chemokines for the activation of cytotoxic T cells, which attract more cytotoxic T cells to infiltrate into tumor tissues and exert anti-tumor effects (Gandhi et al., 2021). We found that inhibition of GPR43 with GLPG0974 significantly diminished the expression of CXCL9, CXCL10 and CXCL11 in OPC tissues from acetate and 4NQO-treated mice, leading to a reduction in the infiltration of CD8^+^ T cells and promoting the OPC progression (Figures 7D–H). In brief, the *Lactobacillus* metabolites enhanced the expression of IFN-*γ* and IFN-*γ*-inducible chemokines in tumor tissues by activating GPR43, thereby promoting the infiltration of CD8^+^ T cells and inhibiting the OPC development (Figure 8).

## Discussion

4NQO, a precursor carcinogen, is an aromatic amine heterocyclic compound, which is metabolized in two pathways in the body. The first metabolic pathway is the formation of near-carcinogen 4-hydroxyaminoquinoline-1-oxide through the catalyzation of 4NQO reductase, which is further metabolized into final carcinogen 4-acetylaminoquinoline-1-oxide by prolylation. Finally, it binds to the nucleophilic structure of the target organ’s DNA to form DNA admixture, leading to G→A transformation in the 12th codon of H-ras gene on mouse chromosome 7 and chromosome damage (Makita et al., 1996). The second pathway is the detoxification of 4NQO, which forms glutathione (GHT) conjugates by glutathione transferase S (GSTs) in the body (Li et al., 1997). Imbalance of the two metabolic pathways is the main reason that 4NQO induces tumors. 4NQO reductase plays a key role in the carcinogenic process of 4NQO. Tanaka et al. reported that 4NQO reductase bears the highest content in the glossopharynx mucosa of mice (Tanaka et al., 1997). We induced tumors in mice at 8 weeks old and formed tumors 16 weeks after induction. This process spans the animal growth period (4–8 weeks after birth), sexual maturity, body maturity and middle and old age, and the tumorigenesis process is long, similar to the natural course of human OPC. We induced OPC with 4NQO in BABL/c mice, and the severity of the lesions increased gradually with the prolongation of 4NQO treatment. It has undergone the pathological process of mild dysplasia, moderate dysplasia, severe dysplasia, carcinoma *in situ*, and early invasive carcinoma, indicating that the pathological process and results of the oropharyngeal mucosa in mice are similar to human OPC. The 12-week and 16-week exposure are corresponded to the early and middle-advanced stages of the mucosal lesions respectively, with a consistency in lesions among individuals. Thus, the model should be able to successfully reflect the early and middle-advanced stages of OPC.

The oral cavity is one of the microbial reservoirs of the body. About 700 kinds of microorganisms are colonized to maintain the microecological balance in the oral cavity (Zhang et al., 2019). If the homeostasis is broken, it will cause a variety of oral diseases, such as caries, periodontal disease, oral cancer, etc. (Aas et al., 2005; Lamont et al., 2018). Besides, it is also associated with diseases other than oral cavity and oropharyngeal disorders, such as cardiovascular diseases, pancreatic cancer, Alzheimer’s disease, etc. (Koren et al., 2011; Lee et al., 2017; Gaiser et al., 2019). Microorganisms are involved in pathogenesis of multiple cancers, such as *H. pylori* in gastric cancer (Mager, 2006), *Chlamydia trachomatis* in cervical cancer, and *Fusobacterium nucleatum* in colon cancer (Kostic et al., 2012). The role of oral flora in the development and progression of OPC is still unclear. In this study, we found that the diversity and composition of the oral microbiota in OPC mice were significantly different from those in the control mice through 16S rRNA sequencing and bioinformatics analysis. *Firmicutes*, *Proteobacteria*, and *Bacteroidetes* are three phyla with the highest abundance in the mice oral cavity, which constituted the dominant community, according with previous studies (Pushalkar et al., 2011; Guerrero-Preston et al., 2016). The relative abundance of *Firmicutes* decreased in OPC, whereas the *Proteobacteria* increased significantly. Compared with the control mice, OPC mice showed an abundance in *Streptococcus*, *Veillonella*, *Muribacter*, *Rodentibacter*, and *Gemella*, and a decline in *Lactobacillus*. *Lactobacillus* was the dominant genus, which gradually decreased with the disease severity, indicating that the genus may be closely related to OPC.

With the gradual aggravation of dysplasia, the diversity and abundance of the oral flora in OPC mice were significantly higher than those in control mice. The reason for the alteration in OPC mice may be due to the lessen of *Lactobacillus*, the dominant genus, which leads to the colonization of a variety of uncommon conditional pathogens, causing the increase of the diversity and abundance of oral bacteria. Interestingly, Guerrero-Preston et al. collected saliva samples from patients with oral squamous cell carcinoma and found that the diversity of the microbial community in the saliva of patients with oral squamous cell carcinoma was significantly reduced compared to healthy subjects (Guerrero-Preston et al., 2016). In another study, Hu et al. collected saliva samples from oral squamous cell carcinoma and found that microorganisms in the saliva of these patients were more diverse than those in healthy subjects (Hu et al., 2016). The types of microorganisms in saliva are greatly affected by factors such as saliva flow rate, secretion volume, and pH value, which may contribute to the difference in diversity and abundance of oral bacteria. We detected the microbial colonization of glossopharyngeal tissue, including bacteria in the mucosa and saliva, with a wider detection range and more representative. The microbial community structure in OPC mice was significantly different from that in the control and oral microbiota dysbiosis existed in mice with OPC.

Tumor-bearing mice showed significant decline in *Lactobacillus*, as compared with control mice. We further demonstrated that the reduction of *Lactobacillus* played an important role in the occurrence and development of OPC. We treated the mice with *Lactobacillus* and 4NQO simultaneously for 16 weeks, and observed that the carcinogenesis of the mouse glossopharyngeal tissue was significantly suppressed (Figure 3C), indicating that supplementation of *Lactobacillus* could slow down the cancerous process of OPC.

Microbiota regulates host immunity and hence promotes anti-tumor response. CD8^+^ T cells is a critical effector for anti-tumor immunity. However, it remains unclear whether microbiota directly regulates the function of anti-tumor cytotoxic CD8^+^ T cells. In the current study, we demonstrated that microbiota directly promotes anti-tumor CD8^+^ T cell immunity, and *Lactobacillus* slowed down the OPC process through its metabolites, especially acetate. SCFAs, such as acetate, can bind to GPR43, with varying affinities to promote cellular effects in metabolism or changes in immune function (Docampo et al., 2021). In addition, GPR43 promotes the expression of IFN (Antunes et al., 2019). IFN-regulated gene transcription is increased and correlates with the extent of immune cell infiltration, indicating that IFN-related immune responses play an important role in immune surveillance (Wenzel et al., 2008). It is known that the supernatant derived from tumors can increase the frequency of CD8^+^ T cells that produce IFN-*γ* (Khou et al., 2020). IFN-*γ*-inducible CXCL9, CXCL10 and CXCL11 are chemokines for cytotoxic T cells, which attract more cytotoxic T cells to infiltrate into tumor tissues and exert anti-tumor effects (Gandhi et al., 2021). Interestingly, we found that acetate upregulated the transcription and expression of GPR43, accompanied by an increase in the production of IFN-*γ* and IFN-*γ*-inducible CXCL9, CXCL10 and CXCL11, enhanced the infiltration of CD8^+^ T lymphocytes, and delayed tumorigenesis of OPC, suggesting that acetate generated by microorganisms could promote anti-tumor immunity to sufficiently improve the therapeutic efficacy. Therefore, supplement of probiotics or application of microbial metabolites could be an essential procedure for the improvement of anti-tumor immunity. Whether microbiota directly or indirectly regulates the anti-tumor CD8^+^ T cell response or other microbial metabolites participated in the anti-tumor immunity in OPC needs further research.

## Data Availability

The authors declare that all the data supporting the findings of this study are available within the paper and its Supplementary Materialfiles.

## Supplementary Material

Supplementary Figure S1 Precancerous lesions and OPCs in mice treated with 4NQO. Healthy BALB/c male mice were housed in SPF environment in Henan University on a 12 h light-dark cycle with food and water available ad libitum. The mice were randomly divided into experimental group (*n* = 20) provided with drinking water containing 100 mg/L 4NQO (sigma, United States) and into control group (*n* = 20) provided with drinking water without 4NQO. Five mice were randomly killed every 4 weeks continuously for 16 weeks. **(A)** Experimental process line. **(B)** Body weight of mice. **(C)** HE staining of glossopharyngeal tissue at different time points. Data represent mean ± SEM. **p* < 0.05, *n* = 5, compared with control.

Supplementary Figure S2 SCFAs levels in the serum of mice treated with *Lactobacillus* culture supernatant. *Lactobacillus* culture supernatant (Lac-Medium) or Medium alone was dripped into mouth of mice treated with 4NQO for 16 weeks. SCFAs in murine serum were quantified as described in Materials and Methods. **(A–G)** Levels (µg/ml) of acetate, propionate, isobutyrate, butyrate, isovalerate, valerate, and caproate in sera of the mice. Data represent mean ± SEM. * *p* < 0.05, *n* = 5, compared with Medium.
