# Supplementary figures and images for: Lactobacillus Suppresses Tumorigenesis of Oropharyngeal Cancer via Enhancing Anti-Tumor Immune Response
Source: Front Cell Dev Biol. 2022 Mar 1;10:842153. doi: 10.3389/fcell.2022.842153 (PMC8920992; doi:10.3389/fcell.2022.842153)

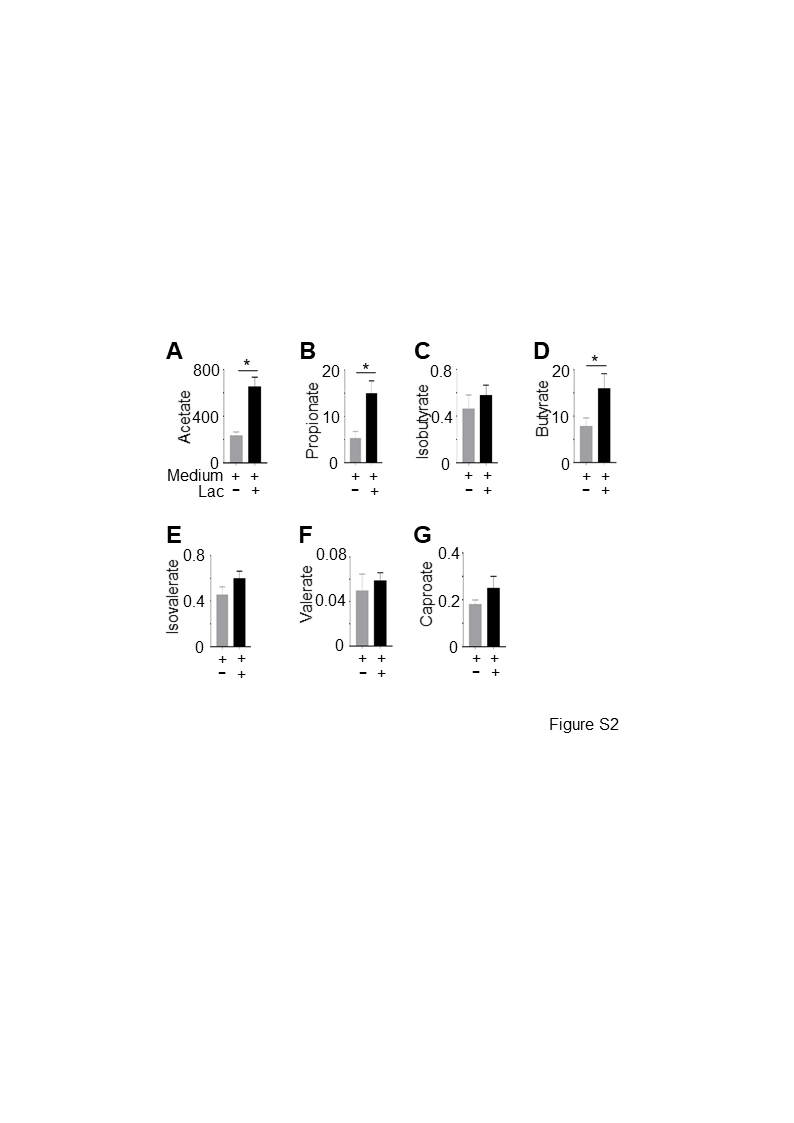

Supplement: Supplementary file 1 [file Image2.TIF]

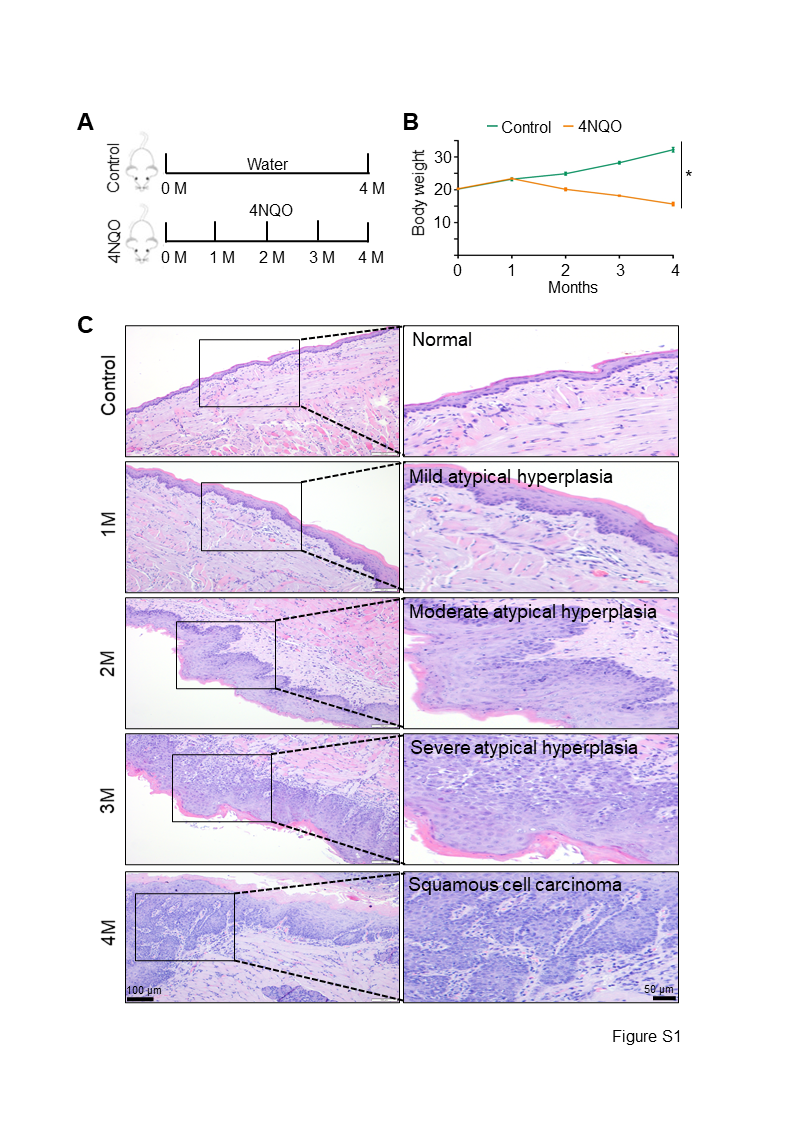

Supplement: Supplementary file 2 [file Image1.TIF]
